# Stratification of Pediatric COVID-19 Cases Using Inflammatory Biomarker Profiling and Machine Learning

**DOI:** 10.3390/jcm12175435

**Published:** 2023-08-22

**Authors:** Devika Subramanian, Aadith Vittala, Xinpu Chen, Christopher Julien, Sebastian Acosta, Craig Rusin, Carl Allen, Nicholas Rider, Zbigniew Starosolski, Ananth Annapragada, Sridevi Devaraj

**Affiliations:** 1 Department of Computer Science, Rice University, 6100 Main St. MS 132, Houston, TX 77005, USA; 2 Texas Children’s Hospital/Baylor College of Medicine, 6621 Fannin Street, WB110.06, Houston, TX 77030, USA

**Keywords:** COVID-19, SARS CoV-2, inflammation, cytokine, machine learning

## Abstract

While pediatric COVID-19 is rarely severe, a small fraction of children infected with SARS-CoV-2 go on to develop multisystem inflammatory syndrome (MIS-C), with substantial morbidity. An objective method with high specificity and high sensitivity to identify current or imminent MIS-C in children infected with SARS-CoV-2 is highly desirable. The aim was to learn about an interpretable novel cytokine/chemokine assay panel providing such an objective classification. This retrospective study was conducted on four groups of pediatric patients seen at multiple sites of Texas Children’s Hospital, Houston, TX who consented to provide blood samples to our COVID-19 Biorepository. Standard laboratory markers of inflammation and a novel cytokine/chemokine array were measured in blood samples of all patients. Group 1 consisted of 72 COVID-19, 70 MIS-C and 63 uninfected control patients seen between May 2020 and January 2021 and predominantly infected with pre-alpha variants. Group 2 consisted of 29 COVID-19 and 43 MIS-C patients seen between January and May 2021 infected predominantly with the alpha variant. Group 3 consisted of 30 COVID-19 and 32 MIS-C patients seen between August and October 2021 infected with alpha and/or delta variants. Group 4 consisted of 20 COVID-19 and 46 MIS-C patients seen between October 2021 andJanuary 2022 infected with delta and/or omicron variants. Group 1 was used to train an L1-regularized logistic regression model which was tested using five-fold cross validation, and then separately validated against the remaining naïve groups. The area under receiver operating curve (AUROC) and F1-score were used to quantify the performance of the cytokine/chemokine assay-based classifier. Standard laboratory markers predict MIS-C with a five-fold cross-validated AUROC of 0.86 ± 0.05 and an F1 score of 0.78 ± 0.07, while the cytokine/chemokine panel predicted MIS-C with a five-fold cross-validated AUROC of 0.95 ± 0.02 and an F1 score of 0.91 ± 0.04, with only sixteen of the forty-five cytokines/chemokines sufficient to achieve this performance. Tested on Group 2 the cytokine/chemokine panel yielded AUROC = 0.98 and F1 = 0.93, on Group 3 it yielded AUROC = 0.89 and F1 = 0.89, and on Group 4 AUROC = 0.99 and F1 = 0.97. Adding standard laboratory markers to the cytokine/chemokine panel did not improve performance. A top-10 subset of these 16 cytokines achieves equivalent performance on the validation data sets. Our findings demonstrate that a sixteen-cytokine/chemokine panel as well as the top ten subset provides a highly sensitive, and specific method to identify MIS-C in patients infected with SARS-CoV-2 of all the major variants identified to date.

## 1. Introduction

Severe Acute Respiratory Syndrome Coronavirus 2 (SARS-CoV-2) infection is typically milder in children than in adults, yet a significant number of patients still need hospitalization [1]. A life-threatening consequence of the infection in children is multisystem inflammatory syndrome (MIS-C). The WHO definition of MIS-C describes this condition occurring in patients under 19 years of age, presenting within 12 weeks following SARS-CoV-2 primary infection or exposure with high fever for at least 3 days, two or more clinical features, disease activity and at least one elevated laboratory marker of inflammation [2]. While the risk factors that predispose some children to develop MIS-C are not fully understood, identification of MIS-C is important because severe organ dysfunction and death have been reported in these patients [3,4]. A prediction of MIS-C at initial presentation enables early initiation of immunotherapies that reduce severity and improve outcomes. Acute respiratory distress syndrome, mainly triggered by acute uncontrolled release of pro-inflammatory cytokines, and referred to as cytokine storm, is a leading cause of severity and morbidity in SARS-CoV-2 infected patients, and a recent meta-analysis of studies in adults [5] suggest that the cytokine storm in SARS-CoV-2 infected patients is directly linked to disease severity. In this study, we therefore aimed to characterize the cytokine/chemokine profile of pediatric patients with MIS-C compared to those with SARS-CoV-2 infection (COVID-19) and to derive a method to stratify pediatric patients presenting with COVID-19 by their risk of MIS-C.

## 2. Subjects and Methods

### 2.1. Study Design

Serum and plasma samples were obtained from the Texas Children’s Hospital COVID-19 Biorepository (TCB) established in April 2020 under protocol H-48474 approved by the Institutional Review Board at Baylor College of Medicine. This repository holds serum/plasma samples from patients admitted with COVID-19 and/or MIS-C to any of the sites of the Texas Children’s Hospital (TCH) system. In addition, samples from patients with no known inflammatory condition were included as controls. The TCH system serves the greater Houston metropolitan area of about 10,000 square miles, which incorporates 9 counties with a combined population of 8.2 million. This study used a training cohort of 205 patients who presented at one of the sites of TCH between April 2020 and January 2021. Of this cohort, 70 had a diagnosis of MIS-C by the CDC criteria, 72 were diagnosed with COVID-19 but did not develop MIS-C, and 63 were controls. The training cohort was obtained when the pre-alpha strain of the SARS-CoV-2 virus was predominant in the population. Three additional validation sets were also obtained. Validation cohort 1 constituted 92 new patients (43 MIS-C, 29 COVID-19 and 20 controls) treated in the TCH system between January 2021 and May 2021. Validation cohort 2 constituted 78 new patients (32 MIS-C, 30 COVID-19 and 16 controls) treated in the TCH system between August 2021 and October 2021. Validation cohort 3 had 76 patients (20 COVID-19, 46 MIS-C and 10 controls) treated in the TCH system between October 2021 and January 2022. While the variants infecting the specific patients from whom these samples were drawn were not recorded, the distribution of strains in TCH system patients as a function of time was recorded (Supplementary Figure S1a,b). Based on this temporal distribution, we infer that the training cohort were infected with predominantly pre-alpha variants, validation cohort 1 predominantly alpha, validation cohort 2 predominantly delta and validation cohort 3 predominantly delta and omicron variants of SARS-CoV-2. Supplementary Figure S2 shows the distribution of time intervals between a patient receiving diagnosis of MIS-C and the time of blood sampling, where negative times indicate that the sample was drawn after the diagnosis had already been made, while positive times indicate that the patient received a diagnosis of MIS-C after the blood sample was drawn. Of note is that roughly half of the MIS-C patients had not yet met CDC criteria, nor had they received a diagnosis at the time the sample was drawn.

### 2.2. Methods

#### 2.2.1. Serum Protein/Laboratory Data/EHR Data Collection

Frozen aliquots of serum/plasma from the TCB were used for biomarker analysis. Values of thirteen clinically used laboratory markers of disease activity and inflammation (C-Reactive Protein (CRP), Procalcitonin, D-Dimer, B-type Natriuretic Peptide (BNP), Sodium, Platelet count, Albumin, Fibrinogen, Protime, Neutrophil to Lymphocyte ratio (NLR), Total CO_2_, Ferritin and Troponin I) were measured since they have been shown previously to be associated with both adult and pediatric SARS-CoV-2 infection [5,6]. These laboratory markers were measured in blood samples collected at the same time as the biorepository samples. Demographic data (age, gender, race and ethnicity), vitals and results of SARS-CoV-2 antibody and PCR testing were all gathered from the patients’ electronic health records (EHR). In addition, specifics of treatments administered (IVIG, anti-IL1RA, steroids, etc.) during the hospital stay were gathered from the EHR. Parameters marking the severity of disease, including length of stay in the hospital, length of stay in the ICU, and use of ventilators, ECMO, oxygen and CPAP were all also obtained from the EHR.

#### 2.2.2. Confirmation of SARS-CoV-2 Infection

All patients whose nasopharyngeal swab tested positive for SARS-CoV-2 using either a transcription-mediated amplification assay or reverse-transcriptase PCR assay were considered confirmed cases of SARS-CoV-2 infection. For MIS-C clinical definition, the Brighton classification by Vogel et al. were applied [2].

#### 2.2.3. Cytokine/Chemokine Profiling

Cytokines/chemokines were analyzed using a 48-plex Millipore MAP Human Cytokine/Chemokine Magnetic Bead Panel (Millipore, St. Louis, MO, USA). Each sample was run in duplicate in separate measurements on a Luminex^®^ MAGPIX instrument. Both kit-derived quality controls (QCs) and an in-house sample pool were used to control for lot-to-lot variability. A total of 45 of the 48 cytokines/chemokines with less than 10% inter- and intra-assay coefficients of variation and with less than 15% difference in duplicate readings were retained for analysis.

#### 2.2.4. Computation

All calculations and computation were performed in, and all algorithms implemented in, Anaconda Python 3.9 with the sklearn and scipy packages.

#### 2.2.5. Univariate Discrimination with the Wilcoxon Rank-Sum Test

The ability of laboratory and cytokine/chemokine analytes to discriminate COVID-19 from MIS-C groups was assessed using the non-parametric Wilcoxon rank-sum test on the training data (72 COVID-19 samples, 66 MIS-C samples). We tested the null hypothesis that there is no difference in the medians of the biomarker values between the COVID-19 and MIS-C cohorts. *p* ≤ 0.05 was used to indicate significant differences in the distributions of the analyte in the two populations.

#### 2.2.6. Multivariate Cross-Validated L1 Regularized Logistic Regression Classification

An L1-regularized logistic regression augmenting the cross-entropy loss function with a penalty term proportional to the sum of the absolute values of the regression coefficients [7,8] was used for supervised learning. Five-fold cross validation was used to ensure generalization performance within the training set, characterized by the area under the receiver operating curve (AUROC) and the F1-score [9].

#### 2.2.7. Multidimensional Data Representation

The Uniform Manifold expression and Projection [10,11,12] (UMAP) technique was used to reduce dimensionality of the dataset and visualize multidimensional cytokine/chemokine data in an unsupervised manner.

#### 2.2.8. Network Analysis

The STRING database [13] of protein–protein interactions including both physical associations and functional associations was used to identify protein interaction networks associated with the cytokine/chemokine combinations characteristic of COVID-19 and MIS-C [14,15,16]. Interactions with confidence score > 0.9 were selected for network construction. In addition, we added connections between any two cytokines/chemokines that had high correlations (Pearson correlation > 0.8) in our training data. To visualize networks of proteins affected by either COVID-19 or MIS-C, we computed subnetworks composed of all cytokines elevated or depressed two-fold or more when comparing the median level for patients with either COVID-19 or MIS-C to the levels in control patients. To place the subnetworks in a more global context, we also added proteins that were not measured into the network if they interacted with at least four measured cytokines/chemokines in the network. Graph construction and visualization were performed using custom-written Python 3.9 scripts.

## 3. Results

### 3.1. Clinical Characteristics of the Training Cohort and Validation Sets

Table 1a summarizes clinical and demographic characteristics for the training cohort, and results of the Wilcoxon rank-sum test testing the null hypothesis that there is no difference in each variable between MIS-C and COVID-19 patients. Table 1b summarizes the same characteristics for validation sets 1, 2 and 3. In the training cohort, significant differences between the MIS-C and COVID-19 groups were noted in median age (9 vs.14 years), sex (62% male vs. 46%), median hospital stay duration (7.8 days vs. 5.5 days), median ICU stay (3.4 days vs. 0 days), ventilator use (30.8% vs. 17.1%) and incidence of acute kidney injury (AKI) (35.3% vs. 11.4%). There were no significant differences in the distribution of race, ethnicity and BMI values in the two cohorts. The demographics of the three validation cohorts mirror that of the training set, except for the COVID-19 cohort of validation set 3 which has only 20 patients. The differences between the COVID-19 and MIS-C cohorts in median hospital stay duration, median ICU stay, ventilator use and AKI that are observed in the training cohort, are preserved in the three validation sets. However, the median values of these quantities decrease over time, which is consistent with evolution of the virus to higher infectivity/lower disease severity, as well as improvements in disease management and use of vaccines prior to validation set 3. For example, the median length of stay in hospital for the COVID-19 group goes down from 5.5 days in the training cohort to 3.8 days in validation sets 1 and 2 to 0.5 days in validation set 3. A corresponding drop is observed in the length of hospital stay for the MIS-C cohort starting at 7.8 days through to 6.7 days, 6.0 days, to 5.8 days in validation set 3. Similar drops over time are seen for median ICU length of stay, ventilator use, CPAP and AKI in both MIS-C and COVID-19 cohorts in the three validation sets.

### 3.2. Laboratory Characteristics of the Training Cohort and Validation Sets

Table 1b also summarizes statistics for all the measured laboratory markers for the training cohort, and Table 1c provides these statistics for validation sets 1, 2 and 3. Markers that were significantly elevated in the MIS-C training cohort compared to the COVID-19 training cohort are CRP (median 15.9 mg/dL, *p* = $5.2\times 10^{-10}$), ferritin (median 333.3 mg/L, *p* = $7.2\times 10^{-3}$), procalcitonin (median 4.7 ng/mL, *p* = $4.6\times 10^{-9}$), fibrinogen (median 513 mg/dL, *p* = $1.8\times 10^{-5}$), protime (median 15.4 s, *p* = $1.6\times 10^{-4}$), D-dimer (median 3.0 µg/mL, *p* = $1.4\times 10^{-7}$), BNP (median 221.6 pg/mL, *p* = $1.5\times 10^{-7}$) and the neutrophil to lymphocyte ratio (NLR), (median 8.0, *p* = $3.3\times 10^{-4}$). Markers that were significantly lowered in the MIS-C training cohort compared to the COVID-19 training cohort are albumin (median 3.3 g/dL, *p* = $9.7\times 10^{-7}$), platelet counts (median 158/µL, *p* = $7.0\times 10^{-7}$) and sodium (median 134, *p* = $2.5\times 10^{-7}$). Troponin I level between the two cohorts was not significantly different.

In the three validation sets, for the MIS-C cohort, ferritin, fibrinogen, protime, D-Dimer, albumin and sodium levels remain about the same as in the training set. However, other inflammation markers improve from the training MIS-C cohort (pre-alpha) to the MIS-C cohort in validation set 3 (delta and omicron variants): platelet counts increase from 158/µL in the training MIS-C cohort to 199/µL in validation set 3, CRP declines from 15.9 mg/dL to 7.9 mg/dL, procalcitonin drops from 4.7 ng/mL to 1.8 ng/mL and BNP decreases from 221.6 pg/mL to 143 pg/mL. In the three validation sets, for the COVID-19 cohort, there are significant changes in four laboratory markers of inflammation from the training group to validation set 3: albumin drops from 4.2 g/dL to 3.6 g/dL (closer to the MIS-C group in the training set), BNP increases from 64 pg/mL to 169 pg/mL, procalcitonin from 1.6 to 0.4 and CRP from 2.1 mg/dL to 13.9 mg/dL. These data are consistent with MIS-C cases getting milder as the virus evolves, and COVID-19 becoming more severe, relative to the initial pre-alpha manifestation. Further, these trends suggest that standard laboratory markers of inflammation change with the severity of the variant/disease course and cannot distinguish between MIS-C and COVID-19 reliably.

### 3.3. Cytokine/Chemokine Profiles of the Training Cohort

Table 1c shows the levels of 45 cytokines/chemokines ordered by the value of p from the Wilcoxon rank-sum univariate test differentiating COVID-19 from MIS-C in the training cohort. A total of 34 of the 45 cytokines/chemokines were statistically significantly different in the univariate sense. The top sixteen markers of these are soluble IL2 receptor, IP-10, MIG, IL-10, IL-15, IL-3, IL-1RA, TNF-α, IL-13, IFN-ϒ, IL-22, IL-2, TGF, GCSF, IL-6, and IL-27 all with *p* < 3 × 10^−6^. Also shown in Table 1c are the specificity and sensitivity of each individual biomarker with five-fold cross-validation on the training data. No single cytokine/chemokine has both specificity and sensitivity over 0.9.

### 3.4. Machine Learning Models Differentiating COVID-19 from MIS-C

Supplementary Figure S3 shows an L1 regularized logistic regression model trained with five-fold cross-validation using the 13 standard lab biomarkers. The model selects markers, omitting Troponin I. Performance statistics of this model on the training set as well as the three validation sets are in Supplementary Table S1. On the training data, the model exhibits a five-fold cross-validated AUC of 0.86 ± 0.05 and an F1 score of 0.78 ± 0.07. On validation set 1 of 29 COVID-19 and 43 MIS-C patients, the model makes 15 errors (5 COVID-19 and 10 MIS-C) with an AUC of 0.85 and an F1 of 0.81. On validation set 2 of 32 COVID-19 and 30 MIS-C patients, the model makes 14 errors (3 COVID-19 and 11 MIS-C) with an AUC of 0.84 and an F1 of 0.75. On validation set 3 of 20 COVID-19 and 46 MIS-C patients, the model makes 16 errors (4 COVID-19 and 12 MIS-C) with an AUC of 0.83 and an F1 of 0.71. ROC curves for the above are also depicted in Supplementary Figure S5.

**Figure 1:**
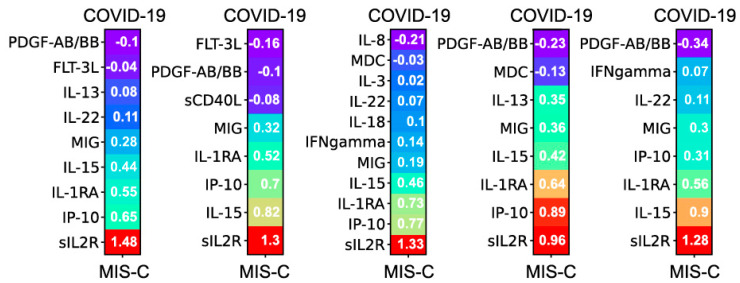
L1 regularized logistic regression model trained with 5-fold cross-validation on the cytokine/chemokine data obtained from the training cohort. The model uses a total of 16 of the available 45 cytokines/chemokines. Each bar is a graphical representation of the five logistic models obtained with 5-fold cross-validation. The coefficients of each model are presented in sorted order. Each coefficient represents the change in log-odds of MIS-C with unit change in the value of that cytokine/chemokine.

Figure 1 shows an L1 regularized logistic regression model trained with five-fold cross-validation on the cytokine/chemokine data obtained from the training cohort. The coefficients of each of the five models are shown in sorted order, with each coefficient representing the change in log-odds of MIS-C corresponding to unit change in the value of the cytokine/chemokine. The model uses a total of 16 of the available 45 cytokines/chemokines to achieve a cross-validated AUC of 0.95 ± 0.02 and F1 score of 0.91 ± 0.04. The performance of L1-regularized models built with both cytokines/chemokines and laboratory biomarkers as predictors was identical to models built with cytokines/chemokines alone. The addition of laboratory biomarkers to the overall set of predictors was therefore determined to not improve the performance of the models.

On validation set 1 of 29 COVID-19 and 43 MIS-C patients, the model makes six errors (0 COVID-19 and 6 MIS-C) with an AUC of 0.98 and an F1 of 0.93. On validation set 2 of 32 COVID-19 and 30 MIS-C patients, the model makes eight errors (5 COVID-19 and 3 MIS-C) with an AUC of 0.89 and an F1 of 0.88. The drop in performance on the second validation set is consistent with the prevalence of the delta variant in this cohort with inflammation in COVID-19 being more severe compared to the pre-alpha training cohort. On the third validation set of 20 COVID-19 and 46 MIS-C patients, the model makes three errors (1 COVID-19 and 2 MIS-C) with an AUC of 0.99 and an F1 of 0.97. The confusion matrices and AUCROC curves for the three validation cohorts are presented in Supplementary Figure S3. Importantly, these results do not change significantly even when the model is restricted to 10 cytokines/chemokines composed of the top five predictive of COVID-19 and the top five predictive of MIS-C from the original 16 biomarker model. These 10 cytokines/chemokines are soluble IL2R, IP-10 (CXCL-10), IL-1RA, IL-15, MIG (CXCL-9), MDC (CCL 22), IL-8, G-CSF, FLT-3L and PDGF-AB/BB. There is some overlap with the cytokines/chemokines reported in Sacco et. al. [17] who find IP-10, IL-2, MDC, IL-15 significant at the univariate level for discriminating MIS-C from COVID-19. However, as shown in Table 1c, none of these markers have high specificity and high sensitivity at an individual level. Our multivariate analysis reveals a combination of 16 (or a subset of 10) with excellent discriminative performance on a large patient cohort gathered over time, as the virus evolved.

**Figure 2:**
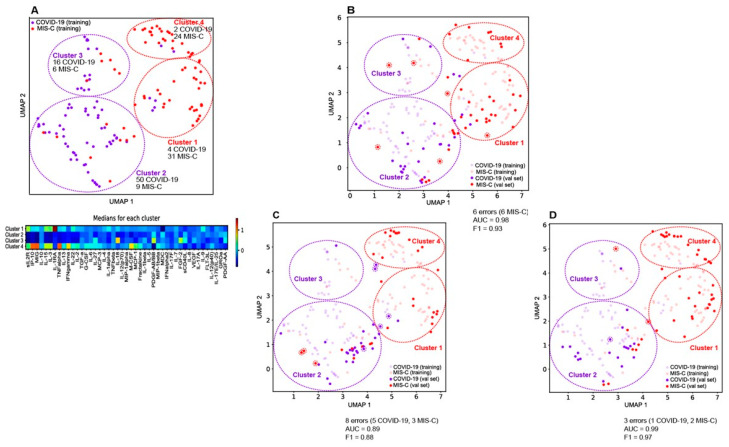
(**A**) Top: a two-dimensional UMAP projection of the 45-dimensional cytokine/chemokine vector representing each sample in our training cohort. The COVID patients in the validation set are colored purple, while the MIS-C patients are colored red. (**A**) Bottom: the medians of key cytokines/chemokines for each of the four clusters. (**B**) The first validation set projected back into the UMAP coordinates derived from the training data. Misclassified COVID-19 and MIS-C patients are called out by dotted circles of purple and red. For the first validation set with 29 COVID and 43 MIS-C, only 6 MIS-C patients were misclassified. Five of them fall in the COVID clusters defined by the training cohort, and these MIS-C patients were confirmed by chart review to be mild cases. (**C**) The second validation set projected back into the UMAP coordinates derived from the training data. Misclassified COVID-19 and MIS-C patients are called out by dotted circles of purple and red. For the second validation set with 32 COVID-19 and 30 MIS-C patients, 5 COVID-19 and 3 MIS-C patients were misclassified. In total, 4 of the COVID-19s fall in the MIS-C clusters defined by the training cohort, showing the evolution of the disease with the COVID-19 patients having more severe disease compared to the initial training cohort. The 3 misclassified MIS-C’s have a mild version of the disease and fall into the low risk COVID-19 cluster defined by the training data. (**D**) The third validation set projected back into the UMAP coordinates derived from the training data. Misclassified COVID-19 and MIS-C patients are called out by dotted circles of purple and red. For the third validation set with 20 COVID and 46 MIS-C patients, 1 COVID-19 and 2 MIS-C patients were misclassified.

To understand the effectiveness of our multivariate model, a two-dimensional UMAP projection of the 45-dimensional cytokine/chemokine vector representing each sample in the training cohort is constructed. Figure 2A (top) shows the UMAP projection. Two clusters become apparent: COVID-19 patients (purple dots) to the left and the MIS-C patients (red dots) to the right. The separation of the two cohorts in a low-dimensional projection of the data explains the excellent cross-validated performance of the logistic regression model. Note that the separation is not perfect—the logistic regression model misclassifies some of the MIS-C patients with a less severe form of the disease, as reflected in the cytokine/chemokine profiles shown in Supplementary Figure S4a,b.

The COVID-19 and MIS-C patients are further stratified into two clusters each. Cluster 2 (50 COVID-19, 9 MIS-C) and Cluster 3 (16 COVID-19, 6 MIS-C) are predominantly COVID-19 clusters, while Cluster 1 (4 COVID-19, 31 MIS-C) and Cluster 4 (2 COVID-19, 24 MIS-C) are predominantly MIS-C clusters. As shown in Figure 2A (bottom), Cluster 3 represents patients in the COVID-19 cohort with higher median levels of inflammation in the measured cytokines/chemokines. Relative to Cluster 2, Cluster 3 patients have elevated median levels of IL-18, IL-27, PDGF-AB/BB, and FGF-2. Cluster 4 represents patients in the MIS-C cohort with higher median levels of IP-10, MIG, TNF-a, and IFN-g, relative to Cluster 1. Cluster 1 is characterized by an elevation of IL-1RA relative to Cluster 4, potentially reflecting treatment with IVIg/steroids/anakinra. The overall risk stratification/disease severity suggested by the UMAP plot is Cluster 4 > Cluster 1 > Cluster 3 > Cluster 2 (highest to lowest risk and disease severity).

Medians and interquartile ranges for the thirteen laboratory biomarkers for each of the clusters are shown in Supplementary Table S2. As expected, the values of the lab markers in these clusters correlate well with the median cytokine/chemokine profiles from Figure 2A. In addition, we computed medians of the length of stay (days), ICU length of stay (days). Cluster 4 and Cluster 1 (the MIS-C clusters) are associated with longer stays in both the ICU and the hospital compared to Cluster 2 and Cluster 3 (the COVID-19 clusters). To assess severity of disease, we computed the fraction of patients on respiratory support (ECMO, Ventilator, CPAP) for the four clusters. Cluster 4 has the highest usage of ECMO, Ventilator and CPAP, while Cluster 2 has the least usage confirming the severity/risk ordering of the COVID-19 and MIS-C patients.

### 3.5. Generalizability of Model to New Validation Sets

Table 2 shows that the model trained on the initial cohort of 72 COVID-19 and 70 MIS-C patients performs well on three new validation sets from local patients. To understand the classification errors made by the model, we project each of the validation sets into the UMAP coordinate frame defined by the training data.

Figure 2B shows Validation set 1 projected on the training data UMAP. Of the twenty-nine COVID and forty-three MIS-C patients in this validation set, only six MIS-C patients were misclassified. Note that all the new COVID-19 patients fall within the COVID-19 clusters 2 and 3 defined by the training data. Five of the six misclassified MIS-C’s fall in the COVID-19 clusters defined by the training cohort, and these MIS-C patients were confirmed by chart review to be mild cases.

Figure 2C shows Validation set 2 projected on the training data UMAP. Of the thirty-two COVID-19 and thirty MIS-C patients, five COVID-19 and three MIS-C patients were misclassified. Four of the COVID-19s fall in the MIS-C clusters defined by the training cohort, consistent with this set containing more delta variant infected patients with severe disease compared to the initial training cohort. The three misclassified MIS-C’s have a mild version of the disease and fall into the low risk COVID-19 cluster defined by the training data.

Figure 2D shows Validation set 3 projected on the training data UMAP. Of the twenty COVID-19 and forty-six MIS-C patients, one COVID-19 and two MIS-C patients were misclassified. An overall milder disease profile is consistent with the accuracy of this classification.

These projections of the validation sets onto the UMAP of the training data are consistent with the cytokine/chemokine measurements yielding highly accurate predictions of MIS-C even as the disease evolved. The 16 cytokine/chemokine model as well as the minimal set of 10 cytokine/chemokines appears to be robust and maintains its accuracy over time.

### 3.6. Network Analysis of the Cytokine/Chemokine Training Data

**Figure 3:**
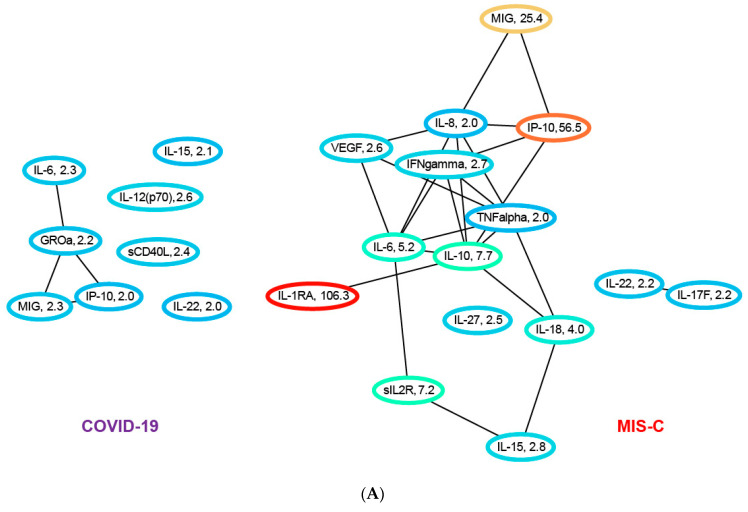

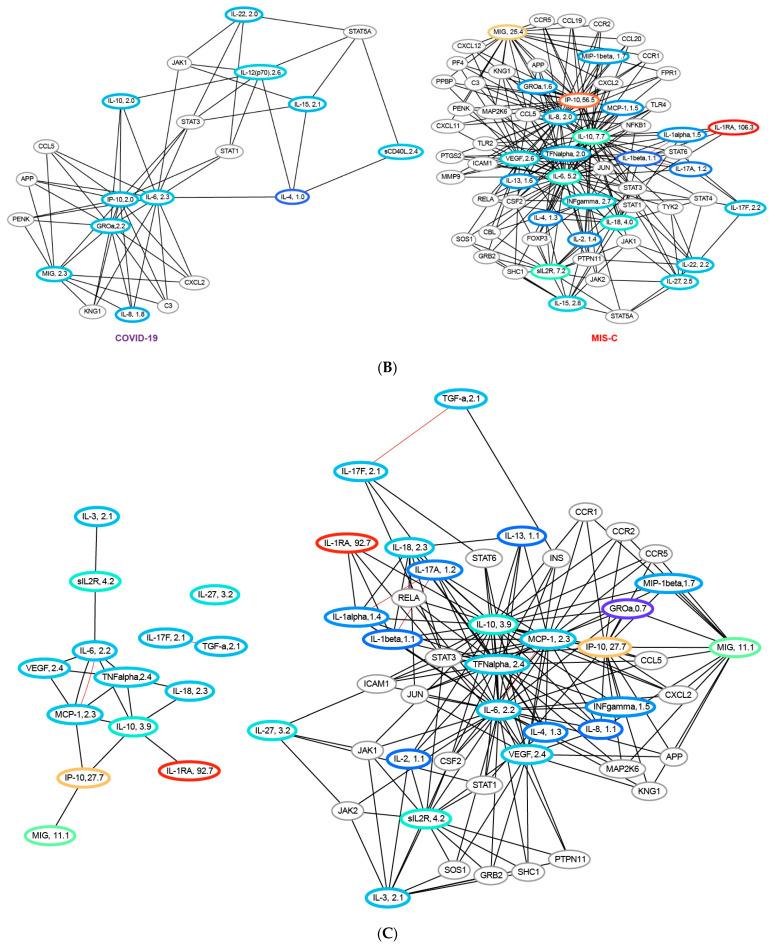
(**A**) Ratio of median cytokine levels in COVID-19 patients to healthy controls and in MIS-C patients to controls for all cytokines/chemokines that are elevated/depressed two-fold or more. The network uses protein–protein interactions (black) from the STRING database to determine connections. Nodes are colored based on the fold change, and the actual fold change is also included in the node label. Many more cytokines/chemokines are inflamed in MIS-C compared to COVID-19. (**B**) Ratio of median cytokine levels in COVID-19 and MIS-C patients to controls for all cytokines elevated or depressed at least two-fold. The network includes non-measured proteins that have protein–protein interactions with at least four measured cytokines. We can see that network dysfunction is more pronounced in MIS-C compared to COVID-19. In the COVID-19 network, we see IL-15, scD40L and IL-12 p(70) connecting to the MIG, IP-10 and IL-6 subnetwork via IL-4. In addition, IL-22 connects back to core subnetwork in COVID-19 via IL-6. In the MIS-C network, IL-22 connects to the core connected component via IL-10. (**C**) (left) Differentially expressed cytokines in MIS-C versus COVID-19 elevated or depressed at least two-fold. Nodes are labeled with the ratio of median cytokine levels in MIS-C to the median cytokine levels in COVID-19. (right) Differentially expressed cytokines in context of other non-measured proteins that have interactions with at least four measured cytokines. The red edge in the left network denotes correlation of 0.8 or more in the training data between the pair of cytokines/chemokines.

The cytokine profiles in MIS-C and COVID-19 are significantly different. Figure 3A shows the elevated cytokines/chemokines in COVID-19 and in MIS-C in a network where the edges denote protein–protein interactions derived from the STRING database. An edge represents either a direct or an indirect (via a longer pathway) protein–protein interaction. Both networks reflect inflammation and immune activation, but the MIS-C network is far more extensive, involving many more cytokines/chemokines displaying orders of magnitude higher levels of inflammation. Compared to controls with no known inflammatory condition, the median levels of eight chemokines/cytokines in the COVID-19 patients in our training cohort exhibit a roughly two-fold elevation over healthy controls: IL-6 (2.3), GROa (2.2), MIG (2.3), IP-10 (2.0), IL-15 (2.1), IL-12 p(70) (2.6), sCD40L (2.4) and IL-22 (2.0). In contrast, compared to healthy controls, the median levels of fifteen chemokines/cytokines in the MIS-C patients are significantly elevated: IL-6 (5.2), MIG (25.4), IP-10 (56.5), IL-15 (2.8), IL-22 (2.2), IL-1RA (106.3), IL-10 (7.7), sIL2R (7.2), IL-18 (4.0), IFN-g (2.7), VEGF (2.6), IL-27 (2.5), IL-17F (2.2), IL-8 (2.0) and TNF-α (2.0). The first five of these cytokines/chemokines are elevated in both the MIS-C and COVID-19 groups, with levels higher in MIS-C than in COVID-19: IL-6 (2.6), MIG (12.7), IP-10 (28.7), IL-15 (1.33) and IL-22 (1.1), where the numbers in parenthesis denote the fold-change in median levels in MIS-C over that in COVID-19 patients. The other ten cytokines/chemokines are elevated only in MIS-C patients.

In both networks, of the cytokines/chemokines we measured, IL-6, MIG and IP-10 contribute the most to the cytokine storm. However, high levels of soluble IL2R, IL-1RA, IL-10, IL-18, IL-8, IFN-g, IL-27, IL-17F and TNF-α appear unique to MIS-C, and possibly serve as markers for disease severity. It is interesting to note that the top ten cytokines/chemokines selected by the robust L1-regularized logistic model for differentiating COVID-19 from MIS-C include MIG, IP-10 and IL-15, which are three of the five cytokines/chemokines elevated in both diseases, with significantly greater elevation in MIS-C. The biomarkers sIL2R, IL-1RA and IL-8 are elevated only in MIS-C. Of the other four biomarkers included in the model MDC (1.6) and PDGF-AB/BB (1.4) are elevated in COVID-19 relative to MIS-C, while G-CSF (2.36) and FLT-3L (1.1) are elevated in MIS-C.

To understand the role of elevated cytokines/chemokines in the context of other unmeasured proteins, we augmented the network with proteins that interact with at least four of the measured biomarkers. The augmented networks for both COVID-19 and MIS-C are shown in Figure 3B. These longer-range interactions connect the disconnected components in both networks to the core subnetworks. In the COVID-19 network in Figure 3B, IL-15, scD40L and IL-12p70 connect to the MIG/IP-10/IL-6 subnetwork via IL-4. In addition. IL-22 connects to the core subnetwork in COVID-19 in Figure 3 via IL-6. In the MIS-C network, IL-22 connects to the core connected component in Figure 3 via IL-10. Figure 3B demonstrates the essential connectedness of the networks shown in Figure 3, the extent and scope of immune system dysfunction in MIS-C and the signaling pathways affected by the disease.

Examining differential expression of the cytokines/chemokines between COVID-19 and MIS-C patients in the training cohort, Figure 3C shows cytokines/chemokines for which the ratio of the median levels in the MIS-C group to the median level in the COVID-19 group is greater than 2, i.e., a two-fold or more elevation in MIS-C. A total of 14 cytokines/chemokines are observed to be differentially overexpressed: IL-1RA (92.7), IP-10 (27.7), MIG (11.1), sIL2R (4.2), IL-10 (3.9), IL-27 (3.2), VEGF (2.4), TNF-α (2.4), IL-18 (2.3), MCP-1 (2.3), IL-6 (2.2), IL-3 (2.1), TGF-ß(2.1) and IL-17F (2.1). The protein–protein interaction network corresponding to these differentially expressed cytokines/chemokines identifies sIL2R->IL6->IP-10->IL-10->MIG as the network path with the highest inflammation in MIS-C relative to COVID-19. These cytokines/chemokines are therefore potential targets for therapeutic intervention. Of note is that one of the direct branches of this pathway with extremely high differential expression (IL-1RA) in MIS-C is the target of the drug anakinra, currently used to treat this condition. Figure 3C also shows the differentially expressed cytokines/chemokines in the context of other proteins in the STRING database, revealing additional signaling pathways that could be targeted for therapeutics.

## 4. Discussion

The SARS-CoV-2 virus infects children and is typically mild in most of them, yet a small cohort develops the far more serious condition MIS-C three or more weeks after virus exposure. This work develops and validates an objective diagnostic/prognostic method for MIS-C at initial presentation. Previous attempts to do so include a recently published meta-analysis of 787 MIS-C patients from 21 different studies [14], which identified laboratory markers including lower platelet counts, higher CRP, D-dimer, leukocyte counts and ferritin correlated with MIS-C. Our data demonstrate that standard laboratory markers of inflammation change with the severity of the variant/disease course and cannot distinguish between MIS-C and COVID-19 reliably. Acute respiratory distress syndrome, mainly triggered by acute uncontrolled release of pro-inflammatory cytokines, and referred to as cytokine storm, is a leading cause of severity and morbidity in SARS-CoV-2 infected patients, and a recent meta-analysis of studies in adults [5] suggest that the cytokine storm in SARS-CoV-2 infected patients is directly linked to disease severity. However, there has been no predictive model using these cytokines/chemokines that are elevated in COVID-19 to distinguish between MIS-C and SARS-CoV-2 infection in such a large cohort of patients. We therefore studied novel cytokine/chemokine markers that enable longitudinal monitoring and provide accurate patient stratification. The cytokine/chemokine markers interpreted by a comprehensive machine-learning model provide highly accurate identification of COVID-19 patients with, or at risk of developing, MIS-C. Such identification enables early intervention with IVIg/steroids. The model was trained exclusively on the May 2020 to January 2021 cytokine/chemokine data of 203 patients with COVID-19 and MIS-C, one of the largest such cohorts studied, and predicts MIS-C with a five-fold-cross-validated AUC of 0.95 ± 0.02 and an F1 score of 0.91 ± 0.04. The cytokine-based model uses the levels of sixteen cytokine/chemokine markers to achieve this performance. We validated this model on three new data sets—the first with 72 patients (29 COVID-19, 43 MIS-C) seen between January 2021 and May 2021 infected predominantly of the alpha strain, the second with 62 patients (30 COVID-19, 32 MIS-C) seen between August 2021 and October 2021 with a mix of the alpha and delta strains, and the third with 66 patients (20 COVID-19 and 46 MIS-C) seen between October 2021 and January 2022, predominantly with the delta and omicron strains. The model exhibits an AUC of 0.98 and F1-score of 0.93 on the first validation data set, an AUC of 0.89 and an F1 score of 0.89 on the second validation data set, and an AUC of 0.99 and F1 score of 0.97 on the third validation dataset. A top ten subset of these sixteen cytokines/chemokines achieves equivalent performance on the validation data sets: AUC of 0.99 and F1 score of 0.94 on the first set, an AUC of 0.91 and an F1 score of 0.85 on the second set, and an AUC of 0.99 and an F1 score of 0.97 on the third set. These top ten cytokines/chemokines are sIL2R, IP-10, IL-1RA, IL-15, MIG, MDC, IL-8, G-CSF, FLT-3L and PDGF-AB/BB. Our results form the basis of a new rapid multiplex assay for risk stratification of patients infected with the SARS-CoV-2 virus. The ability of the model trained on the pre-alpha variant to predict MIS-C with high AUC and F1 scores on new validation sets gathered as the virus evolved affirms its robustness and generalizability to new variants as well as to new populations.

UMAP visualizations of the high-dimensional cytokine/chemokine data separates COVID-19 from MIS-C patients well, consistent with the accuracy of the L1-regularized logistic regression model. Clustering of the UMAP plots reveal finer-grained subsets, sorting MIS-C and COVID-19 patients into two groups each based on severity, providing insights into the cytokine profiles of mild vs. severe disease. By projecting the new validation data sets into the UMAP coordinate frame defined by the training data (pre-alpha strain), it is possible to track the evolution of COVID-19 and MIS-C through the progression of variants. We observe the drift of more recent MIS-C patients toward the COVID-19 training cohort clusters, and the drift of the COVID-19 patients toward the MIS-C training cohort clusters in Figure 2B–D, reflecting the changes in median laboratory biomarker values in the three validation sets, reported in Table 1b. The clusters derived from the UMAP visualizations significantly correlated to the severity of disease, with Cluster 4 having the highest usage of ECMO, Ventilator and CPAP, while Cluster 2 has the least usage confirming the severity/risk ordering of the COVID-19 and MIS-C patients.

The sixteen-cytokine/chemokine panel as well as the top ten subset generate useful system-level hypotheses about the pathogenesis of MIS-C. By deriving a protein–protein network using the STRING database, with elevated cytokines/chemokines in COVID-19 and MIS-C as nodes, we visualize the affected signaling pathways in both these conditions. Network analysis of the cytokines and chemokines elevated in COVID-19 versus MIS-C reveals major differences in the scope of the inflammatory response. Eight of the forty-five measured cytokines/chemokines are elevated in COVID-19 and fifteen are elevated in MIS-C. Both networks show immune system dysfunction, but the MIS-C network is far more extensive, involving many more cytokines/chemokines displaying orders of magnitude higher levels of inflammation. In both networks, IL-6, MIG and IP-10 contribute the most to the cytokine storm. However, high levels of soluble IL2R, IL-1RA, IL-10, IL-18, IL-8, IFN-ϒ, IL-27, IL-17F and TNF-α appear unique to MIS-C and could serve as markers for disease severity.

The immunological features of pediatric COVID-19 and MIS-C are the subject of numerous investigations [15,16,17,18,19,20,21,22,23,24,25]. Our patient cohort is one of the largest that has been studied to date, and our set of 45 cytokine/chemokines is one of the most comprehensive panels to be analyzed. Amongst the different investigators who have studied differences between COVID and related diseases and MIS-C, one of the first to report clear differences between KD and MIS-C using IL-6, IP-10 and IL-17A was a small study [25] of 13 MIS-C patients. Recently, other studies [16,17,18,19,26,27,28], have also shown differential cytokine/chemokine expression in MIS-C compared to either controls, COVID-19 or Kawasaki disease. In a small cohort [29] of seven MIS-C patients, differentiation between COVID-19 and MIS-C patients was achieved using a combination of IL-10, IL-1RA, IL-18, IL-6, TNF and IFN-gamma. Subsequently, ref. [30] in a larger cohort of 118 subjects, significant elevations in IL-6, IL-10, IL-17A and IFN-gamma were demonstrated, and correlated to length of hospital stay.

Network analysis reveals that the top ten cytokines/chemokines selected by the robust L1-regularized logistic model for differentiating COVID-19 from MIS-C include a subset (MIG, IP-10 and IL-15) which are three of the five cytokines/chemokines elevated in both conditions, with significantly greater elevation in MIS-C. The biomarkers sIL2R, IL-1RA and IL-8 are elevated only in MIS-C. Of the other four biomarkers included in the model, MDC (1.6) and PDGF-AB/BB (1.4) are elevated in COVID-19 relative to MIS-C, while G-CSF (2.36) and FLT-3L (1.1) are elevated in MIS-C, consistent with the model’s robust performance across a range of de novo validation sets gathered even as the disease itself evolved. The L1-regularized logistic regression model based on the measurement of as few as 10 novel cytokine/chemokine analytes provides a highly sensitive and specific method to predict MIS-C at initial presentation of SARS-CoV-2 infected patients, while also providing key insights into potential therapeutic targets.

## 5. Conclusions

Several investigations have examined different laboratory markers of inflammation, disease activity and cytokine/chemokine signatures for MIS-C and COVID-19. These studies suggest that there is no single laboratory or cytokine/chemokine biomarker that can differentiate MIS-C and COVID-19. This motivated our quest for a multianalyte profile with algorithmic interpretation. Our model which was derived from a pre-alpha strain training cohort is accurate in predicting disease status for all major COVID variants to date (alpha, delta, omicron) in large validation cohorts, and it identifies MIS-C with few errors. The entire model or summary of this study is depicted in Supplementary Figure S6, from the training cohort to the L1 regularized logistic regression models, including why it gives an excellent prediction of MIS-C in subsequent cohorts and plausible mechanistic pathways. With the change in the course and severity of the disease as well as its management with time, it is important to note that standard laboratory markers do not add any significant information to our model. Notably, the model appears to work for both diagnosis and prognosis. Roughly half of the MIS-C patients in this study had not received a MIS-C diagnosis at the time of blood sampling because they had not (yet) met the CDC criteria for MIS-C. In these patients, the model was therefore a prognostic indicator, predicting their diagnosis at a future date. This is important especially because with the advent of the vaccines many of these MIS-C patients that were diagnosed based on presence of SARS-CoV-2 Spike antibodies per definition can no longer be differentiated by the presence of these antibodies since they also mount a robust post-vaccine antibody response.

Unique in our cohorts is the time interval between each subset of patients, reflecting distinct variants of the SARS-CoV-2 virus in those patients, and that our 10-biomarker model provides a highly sensitive and specific method to predict MIS-C at initial presentation of SARS-CoV-2 infected patients. We elucidate plausible pathways for immune dysregulation in MIS-C. A protein–protein interaction network analysis identifies sIL2R, IL6, IP-10, IL-10 and MIG as the network path with the highest inflammation in MIS-C relative to COVID-19, indicating that there are two pathways mediated by NFkB and IFN-ϒ leading to elevated IP-10 and MIG. Understanding the interaction of these pathways as it relates to disease severity in pediatric MIS-C patients is key to providing insights into potential therapeutic targets, as differential responses to SARS-CoV-2 vaccines in this population emerge.

## Figures and Tables

**Table 1:** (**a**) Clinical, demographic and laboratory characteristics of training cohort of 72 COVID and 66 MIS-C patients and validation cohort 1 (29 COVID-19, 43 MIS-C) and cohort 2 (30 COVID-19, 32 MIS-C). Categorical variables are expressed as frequencies or percentages, while continuous quantities are represented as median/interquartile range pairs. The variables that show significant differences between the COVID-19 and MIS-C cohorts at *p* < 0.05, using the Wilcoxon rank-sum test for continuous quantities and the chi-squared test for discrete quantities, are in italics. (**b**) Clinical, demographic and laboratory characteristics of the validation cohort 1 (29 COVID-19, 43 MIS-C), validation cohort 2 (30 COVID-19, 32 MIS-C) and validation cohort 3 (20 COVID-19, 46 MIS-C). Categorical variables are expressed as frequencies or percentages, while continuous quantities are represented as median/interquartile range pairs. (**c**) Cytokines/chemokines ordered by p-value of the Wilcoxon rank-sum univariate test for discriminating COVID-19 from MIS-C in the training cohort (72 COVID-19, 70 MIS-C). A total of 34 of the 45 cytokines/chemokines are statistically significant in a univariate sense. The specificity and sensitivity of each marker in a five-fold cross validation is also shown. All values are in pg/mL.

(a)						
			COVID-19 Training (N = 72)	MIS-C Training (N = 66)		*p*-Value
**Age** at sample collection Med/IQR (yrs)			14 (6–18)	9 (6–14)		*0.013*
**BMI** Med/IQR			20.8 (17.6–30.5)	19.4 (17.1–25.8)		0.4
**Gender**						*0.03*
M/F%			46/54	62/38		
**Race**						0.95
White%			65.7	63.7		
African American%			28.7	28.9		
Asian%			2.8	4.3		
Other%			2.8	2.9		
**Ethnicity**						0.65
Hispanic%			54	49		
not Hispanic%			46	51		
**SARS-CoV-2 test**						*1.3 × 10^−11^*
PCR%			90	0		
Antibody%			10	100		
unknown%			0	0		
**IVIg/steroids** 7 dys before collection. %			33	46		0.08
**IVIg/steroid from collection** days			−8.5 (−41, −1)	0 (−2.75, 1)		*4.9 × 10^−10^*
**LOS in hospital** day			5.5 (2.8, 12.3)	7.8 (5.3, 11.5)		*0.03*
**ICU LOS** days			0 (0, 4.5)	3.4 (1.5, 6.7)		*0.002*
**ECMO**%			1.4	4.6		0.36
**Ventilator**%			17.1	30.8		*0.035*
**CPAP**%			15.7	24.6		0.16
**AKI**%			11.4	35.3		*1.3 × 10^−3^*
**Labs**						
Sodium mEq/L			138 (136, 140)	134 (131, 137)		*2.5 × 10^−7^*
Albumin g/dL			4 (3.4, 4.6)	3.3 (3.0, 3.7)		*9.7 × 10^−7^*
CO_2_ mEq/L			26 (23, 28)	24 (20, 26)		*3.8 × 10^−3^*
BNP pg/mL			64.1 (18.5, 67.5)	221.6 (49.8, 589.2)		*1.5 × 10^−7^*
TropI ng/mL			0.02 (0.01, 0.07)	0.02 (0.01, 0.08)		0.12
Platelets #/nL			280 (176, 404)	158 (123, 240)		*7.0 × 10^−7^*
Protime seconds			14.6 (14, 14.9)	15.4 (14.6, 16.5)		*1.6 × 10^−4^*
D-dimer μg/mL			1.1 (0.5, 2.04)	3.0 (1.7, 4.06)		*1.4 × 10^−7^*
Fibrinogen mg/dL			402 (317, 459)	513 (424, 600)		*1.8 × 10^−5^*
Procalcitonin ng/mL			1.6 (0.1, 2.1)	4.7 (1.6, 12.8)		*4.6 × 10^−9^*
CRP mg/dL			2.1 (0.5, 5.7)	15.9 (5.2, 24.3)		*5.2 × 10^−10^*
NLRatio			2.5 (1.3, 5.4)	8.0 (3.1, 14.6)		*3.3 × 10^−4^*
Ferritin ng/mL			254 (97, 377)	333 (166, 641)		*7.2 × 10^−3^*
**(b)**						
	**COVID-1**9 **Val Set 1 (N = 29)**	**MIS-C** **Val Set 1 (N = 43)**	**COVID-19** **Val Set 2 (N = 30)**	**MIS-C** **Val Set 2 (N = 32)**	**COVID-19** **Val Set 3 (N = 20)**	**MIS-C** **Val Set 3 (N = 46)**
**Age** at sample collect. Med/IQR	13 (7, 19)	9 (6.5, 11)	14 (10, 20)	11 (7, 14)	11.5 (10, 15)	10.5 (7.3, 14)
**BMI** Med/IQR	21.2 (17.8, 28.7)	21.2 (16.9, 23.2)	26.2 (17.9, 32.1)	19.4 (16.2, 21.2)	18.9 (16.5, 31.4)	18.9 (16.4, 21.0)
**Gender**						
M/F%	44/56	58/42	45/55	48/52	56/44	50/50
**Race**						
White%	70.4	65.1	75.9	51.7	67.0	53.0
African American %	22.2	23.3	20.7	27.6	27.0	27.0
Asian%	7.4	7.0	3.4	13.8	6.0	13.0
Other%	0	4.6	0	6.9	0.0	7.0
**Ethnicity**						
Hispanic%	48	56	69	24.1	55.5	66.7
not Hispanic%	52	44	31	75.9	44.5	33.3
**SARS-CoV-2**						
PCR%	100	0	100	0	100	0
Antibody%	0	100	0	100	0	100
unknown%	0	0	0	0	0	0
**LOS in hospital** (days)	3.8 (1.7, 13.2)	6.7(5.6, 8.5)	3.8(1.7, 13.2)	6.0 (5.6, 8.5)	0.5(0.0, 8.0)	5.8 (4.1, 7.7)
**ICU LOS** (days)	0 (0, 3.3)	2.9 (0.8, 4.7)	0 (0, 0)	2.1 (1.3, 3.1)	0(0, 0)	1.9 (0.6, 3.0)
**ECMO** (%)	3.7	4.7	3.4	0.0	0.0	0.0
**Ventilator** (%)	25.9	11.6	13.8	3.4	3.3	3.3
**CPAP** (%)	22.2	39.5	17.2	20.7	10.0	19.0
**AKI** (%)	11.1	30.2	17.2	24.1	10.0	22.6
**Labs**						
Sodium Med/IQR mEq/L	138 (136, 140)	137 (135, 138)	137.5 (136, 139)	134 (132, 137)	137 (136, 139)	134 (130.5, 136)
Albumin Med/IQR g/dL	4.2 (3.4, 4.4)	3.3 (2.8, 3.7)	3.7 (3.1, 4.1)	3.5 (3.0, 3.9)	3.6 (3.1, 3.8)	3.3 (3.0, 3.8)
CO_2_ Med/IQR mEq/L	26 (24, 28)	24 (23, 26)	25.5 (23, 29)	25 (21.5, 26)	26 (23, 28)	24 (20.5, 25)
BNP Med/IQR pg/mL	105.2 (34.2, 169)	259.8 (124, 632)	201 (110, 228)	142.5 (55.3, 636)	169 (100, 231)	143 (65.3, 706)
Trop I Med/IQR ng/mL	0.02 (0.00, 0.03)	0.02 (0.01, 0.07)	0.01 (0.01, 0.01)	0.02 (0.01, 0.08)	0.01 (0.01, 0.01)	0.02 (0.01, 0.08)
Platelets Med/IQR #/nL	264 (194.5, 356)	177 (106, 265)	233.5 (199, 275)	199 (144, 267.5)	230 (198, 279)	199 (145, 270)
Protime Med/IQR sec	15 (14.4, 15.2)	15.1 (14.4, 15.6)	14.2 (13.6, 14.4)	14.9 (14.5, 15.6)	14.4 (13.8, 14.5)	14.9 (14.4, 15.5)
D-Dimer Med/IQR μg/mL	2.4 (1.3, 2.9)	2.9 (1.9, 3.8)	1.6 (0.7, 3.8)	2.8 (1.6, 3.8)	2.8 (0.7, 4.2)	2.7 (1.5, 3.6)
Fibrinogen Med/IQR mg/dL	448 (430, 457)	459 (359, 634)	416 (350, 532)	539 (393, 614)	455 (377, 494)	527 (393, 597)
Procalcitonin Med/IQR ng/mL	1.5 (1.2, 1.6)	4.6 (2.2, 14.2)	0.4 (0.1, 0.5)	2.2 (1.0, 4.8)	0.4 (0.2, 0.5)	1.8 (1.0, 4.8)
CRP Med/IQR mg/dL	3.8 (0.5, 5.5)	19.3 (5.1, 22.8)	13 (4.0, 13.5)	7.9 (4.3, 22.3)	13.9 (4.3, 14.5)	7.9 (4.8, 20.7)
NLRatio Med/IQR	2.3 (1.0, 3.7)	4.7 (2.9, 9.0)	3.7 (2.1, 5.2)	5.7 (2.7, 12.2)	3.5 (2.0, 4.6)	5.7 (2.3, 9.7)
Ferritin Med/IQR ng/mL	239 (100, 253)	282 (182, 472)	211 (38.8, 400)	334 (223.5, 597.5)	277 (44, 592)	302 (224, 532)
**(c)**						
**Cytokine (Pg/mL)**	**COVID-19**		**MIS-C**	***p*** **-Value**	**Specificity**	**Sensitivity**
**sIL2R**	491.8 (388.1, 782.2)		3576.6 (2270.0, 5475.0)	1.97 × 10^19^	0.95 ± 0.07	0.8 ± 0.08
**IP-10**	175.9 (91.4, 494.0)		9000.0 (1991.0, 15, 803.2)	2.57 × 10^17^	0.93 ± 0.05	0.7 ± 0.08
**MIG**	1395.0 (941.6, 2938.5)		27764.5 (12, 618.8, 43, 215.0)	2.71 × 10^16^	0.35 ± 0.43	0.94 ± 0.08
**IL-10**	25.6 (16.2, 59.2)		191.6 (85.3, 368.5)	6.77 × 10^16^	0.87 ± 0.08	0.7 ± 0.15
**IL-15**	15.7 (6.1, 23.8)		39.2 (30.5, 54.2)	4.55 × 10^15^	0.79 ± 0.19	0.79 ± 0.06
**IL-3**	1.2 (0.7, 1.9)		3.3 (2.3, 4.8)	4.19 × 10^14^	0.85 ± 0.1	0.76 ± 0.17
**IL-1RA**	38.7 (21.6, 143.3)		681.2 (155.9, 7816.2)	8.13 × 10^13^	0.9 ± 0.13	0.54 ± 0.13
**TNFalpha**	57.1 (44.3, 85.7)		132.3 (88.7, 225.6)	1.84 × 10^11^	0.8 ± 0.2	0.59 ± 0.21
**IL-13**	42.9 (37.4, 57.5)		63.7 (56.5, 74.3)	8.45 × 10^1^	0.77 ± 0.14	0.64 ± 0.14
**IFNgamma**	7.0 (5.7, 10.8)		17.2 (9.8, 32.0)	4.47 × 10^9^	0.84 ± 0.21	0.37 ± 0.21
**IL-22**	80.1 (68.2, 105.2)		109.8 (94.5, 125.2)	5.15 × 10^8^	0.73 ± 0.18	0.6 ± 0.26
**IL-2**	3.7 (3.1, 4.7)		5.0 (4.3, 5.9)	1.50 × 10^7^	0.78 ± 0.15	0.56 ± 0.22
**TGF-a**	11.5 (8.0, 15.8)		18.4 (14.0, 25.1)	5.75 × 10^7^	0.82 ± 0.08	0.46 ± 0.1
**G-CSF**	66.1 (46.2, 116.1)		155.7 (78.8, 339.3)	6.64 × 10^7^	0.92 ± 0.05	0.31 ± 0.12
**IL-6**	9.4 (4.5, 27.3)		48.4 (12.7, 188.8)	1.12 × 10^6^	0.89 ± 0.07	0.41 ± 0.12
**IL-27**	2216.5 (1368.8, 3763.5)		4241.5 (3043.2, 8846.8)	3.05 × 10^6^	0.0 ± 0.0	1.0 ± 0.0
**MCP-3**	48.4 (41.0, 57.6)		62.3 (49.2, 88.6)	4.14 × 10^6^	0.83 ± 0.21	0.49 ± 0.22
**IL-4**	3.3 (2.1, 4.6)		5.1 (3.5, 7.2)	8.27 × 10^6^	0.85 ± 0.16	0.31 ± 0.17
**IL-1alpha**	27.2 (22.8, 39.3)		39.0 (31.8, 50.8)	1.77 × 10^5^	0.79 ± 0.18	0.31 ± 0.1
**TNFbeta**	15.0 (12.4, 18.5)		18.8 (16.0, 22.3)	1.93 × 10^5^	0.75 ± 0.13	0.34 ± 0.17
**IL-18**	88.6 (54.2, 174.6)		180.5 (128.8, 380.0)	3.38 × 10^5^	0.8 ± 0.12	0.37 ± 0.08
**IL-12(p70)**	8.8 (7.5, 11.9)		10.6 (9.4, 14.3)	4.03 × 10^5^	0.75 ± 0.14	0.39 ± 0.17
**MIP-1alpha**	46.7 (40.3, 63.1)		54.9 (48.7, 67.2)	4.87 × 10^5^	0.78 ± 0.25	0.1 ± 0.11
**M-CSF**	709.8 (461.7, 1071.8)		1073.0 (740.7, 1547.2)	5.04 × 10^5^	0.75 ± 0.16	0.5 ± 0.15
**MCP-1**	350.7 (210.6, 689.1)		715.4 (316.7, 1423.0)	1.12 × 10^4^	0.8 ± 0.21	0.49 ± 0.15
**Fractalkine**	191.1 (169.0, 240.4)		244.8 (191.2, 286.6)	1.16 × 10^4^	0.76 ± 0.17	0.49 ± 0.14
**IL-1beta**	20.8 (16.0, 28.3)		26.1 (21.8, 35.1)	2.16 × 10^4^	0.78 ± 0.12	0.34 ± 0.12
**IL-5**	9.2 (6.4, 17.8)		16.8 (9.1, 30.8)	5.81 × 10^4^	0.84 ± 0.17	0.17 ± 0.21
**PDGF-AB/BB**	28685.5 (18, 716.2, 41, 657.2)		21208.0 (11, 836.0, 28, 590.2)	6.20 × 10^4^	1.0 ± 0.0	0.0 ± 0.0
**MIP-1beta**	25.7 (20.2, 40.7)		37.1 (25.8, 48.3)	7.40 × 10^4^	0.75 ± 0.2	0.34 ± 0.15
**MDC**	541.0 (294.7, 835.9)		345.9 (190.7, 583.0)	1.56 × 10^3^	0.54 ± 0.1	0.7 ± 0.17
**IFNalpha2**	84.0 (73.1, 104.2)		91.0 (83.9, 119.8)	9.47 × 10^3^	0.79 ± 0.16	0.3 ± 0.14
**IL-17F**	35.6 (24.2, 76.7)		46.5 (36.6, 96.2)	1.01 × 10^2^	0.81 ± 0.18	0.14 ± 0.19
**IL-7**	6.3 (3.7, 8.9)		7.6 (5.3, 10.6)	2.72 × 10^2^	0.55 ± 0.28	0.37 ± 0.25
**FGF-2**	144.3 (122.7, 192.0)		164.7 (131.7, 183.2)	7.23 × 10^2^	0.66 ± 0.23	0.21 ± 0.2
**sCD40L**	2781.5 (928.0, 4929.8)		1651.0 (923.7, 3796.0)	7.34 × 10^2^	0.68 ± 0.28	0.4 ± 0.33
**IL-8**	23.5 (11.4, 50.8)		26.8 (18.6, 45.0)	8.08 × 10^2^	0.33 ± 0.13	0.59 ± 0.15
**VEGF**	142.7 (74.0, 224.3)		153.9 (114.5, 276.9)	8.81 × 10^2^	0.78 ± 0.19	0.17 ± 0.12
**IL-17A**	17.9 (11.9, 23.0)		17.4 (14.9, 25.6)	1.03 × 10^1^	0.68 ± 0.34	0.21 ± 0.26
**IL-9**	32.6 (26.0, 43.9)		36.8 (26.4, 50.4)	1.65 × 10^1^	0.69 ± 0.18	0.4 ± 0.1
**FLT-3L**	40.5 (24.7, 63.9)		42.7 (32.5, 62.6)	1.80 × 10^1^	0.54 ± 0.29	0.37 ± 0.25
**IL-12(p40)**	110.7 (72.0, 158.8)		107.3 (89.1, 162.6)	2.88 × 10^1^	0.39 ± 0.11	0.63 ± 0.11
**IL-17E/IL-25**	1908.5 (1601.5, 2447.0)		1974.5 (1560.8, 2507.0)	3.75 × 10^1^	0.96 ± 0.09	0.04 ± 0.09
**GROa**	55.4 (41.3, 76.3)		58.7 (38.6, 87.2)	3.98 × 10^1^	0.68 ± 0.17	0.21 ± 0.13
**PDGF-AA**	3404.5 (1553.2, 4519.0)		2811.0 (1384.8, 4426.8)	4.81 × 10^1^	0.56 ± 0.46	0.46 ± 0.46

**Table 2:** Performance of the logistic regression model trained on cytokines/chemokines of the training cohort and tested on three de novo validation sets gathered as the virus evolved in time. Note that the training set performance is judged by five-fold cross-validation, and thus there is a mean and standard deviation associated with each performance measure. The validation sets are evaluated in a standard train/test configuration. A single model is built with all the training data, and the validation sets are evaluated in turn against this model. Hence there is a single number characterizing the performance of the model along each metric.

	AUC	F1	AUPRC	Accuracy
**Training set (72 C, 66 M)**	0.95 ± 0.02	0.91 ± 0.04	0.97 ± 0.01	0.92 ± 0.04
**Val set 1 (29 C, 43 M)**	0.98	0.93	0.99	6 errors (0 C, 6 M)
**Val set 2 (30 C, 32 M)**	0.89	0.88	0.91	8 errors (5 C, 3 M)
**Val set 3 (20 C, 46 M)**	0.99	0.97	0.99	3 errors (1 C, 2M)

C-COVID, M-MIS-C, Val-validation, AUC-Area under the curve.

## Data Availability

All data for the paper will be made available on request.

## Supplementary Materials

The following supporting information can be downloaded at: https://www.mdpi.com/article/10.3390/jcm12175435/s1, Figure S1: (a) SARS-CoV-2 positive case trends at TCH during the COVID-19 pandemic. (b) Temporal changes in circulating SARS-CoV-2 variants in pediatric patients. Figure S2: Distribution of the number of days after sample collection that a diagnosis of MIS-C was made in the MIS-C cohort in the training set, and validation sets 1 and 2. Figure S3: 5-fold cross-validated L1 regularized model logistic trained by cross-validation using lab biomarker data only. Figure S4: (a) Sorted heat map of 45 measured cytokines/chemokines for the MIS-C and COVID-19 patients in the training set. (b) Sorted heat map of 45 measured cytokines/chemokines for the MIS-C and COVID-19 patients in the training set, with classification errors made by the model on the training set itself. Figure S5: ROC curves and confusion matrix for the training cohort, as well as the three validation cohorts. Figure S6: Flow chart summary. Table S1: Performance of logistic regression model trained on laboratory biomarkers of the initial cohort and tested on three de novo validation sets. Table S2: Characterizing cytokine/chemokine derived UMAP clusters by lab and hospital data.
