# Supplementary material for: Stratification of Pediatric COVID-19 Cases Using Inflammatory Biomarker Profiling and Machine Learning
Source: J Clin Med. 2023 Aug 22;12(17):5435. doi: 10.3390/jcm12175435 (PMC10487951; doi:10.3390/jcm12175435)
Supplement: Supplementary file 1 [file jcm-12-05435-s001.zip › jcm-2491273-supplementary.pdf]

Supplementary Material

Supplementary Figure S1a: SARS-CoV-2 positive case trends at TCH during the COVID-19 pandemic. These data represent the 7-day average of SARS-CoV-2 positive cases over time from March 2020 – February 2022. Specimens from positive cases have been stored at TCH-CB for future studies.

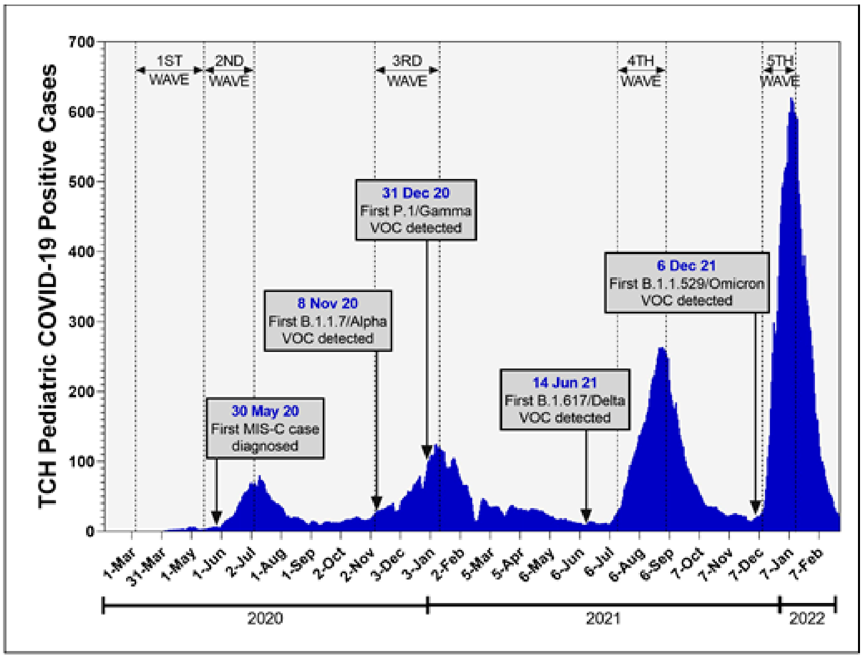

Supplementary Figure S1b: Temporal changes in circulating SARS-CoV-2 variants in pediatric patients. Significant changes have been observed in SARS-CoV-2 variants identified at TCH over the past 22 months. Predominance of lineages have shifted from B.1 in 2020 and B.1.2 in January 2021 to a rapidly shifting variant profile with the emergence of VOCs B.1.1.7 (Alpha), B.1.617/AY.+ (Delta), and BA.+ (Omicron).

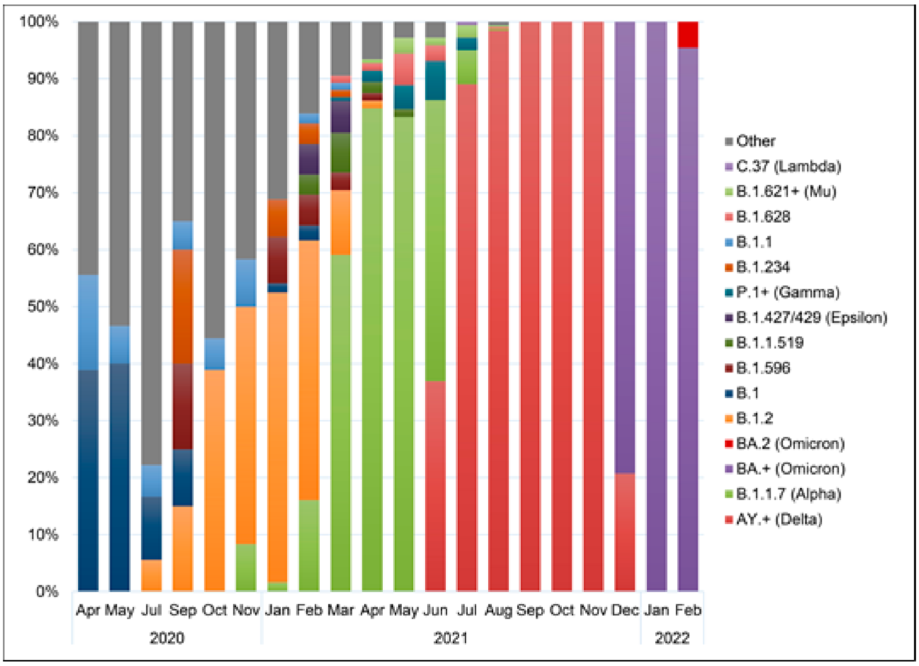

Supplementary Figure S2: Distribution of the number of days after sample collection that a diagnosis of MIS-C was made in the MIS-C cohort in the training set, and validation sets 1 and 2. Negative numbers on the x-axis indicates the sample collection was made after the diagnosis of MIS-C was called. More than half of the MIS-C group had their samples collected prior to diagnosis.

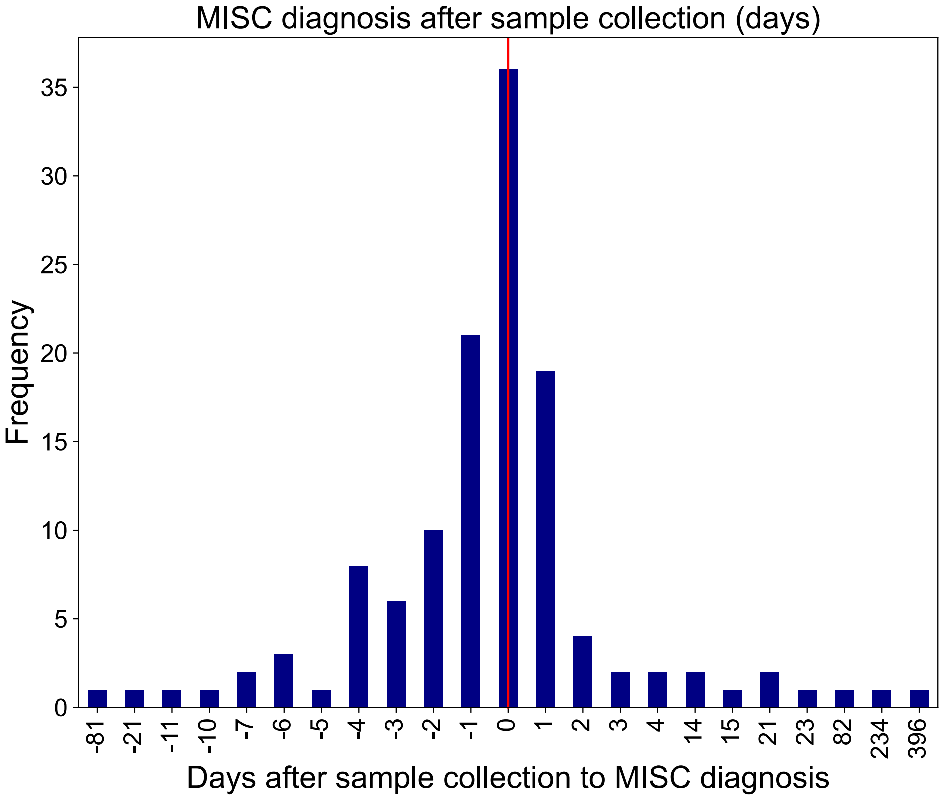

Supplementary Figure S3: 5-fold cross-validated L1 regularized model logistic trained by cross-validation using lab biomarker data only. The model uses a total of 12 lab biomarkers, note that the model does not select Troponin I.

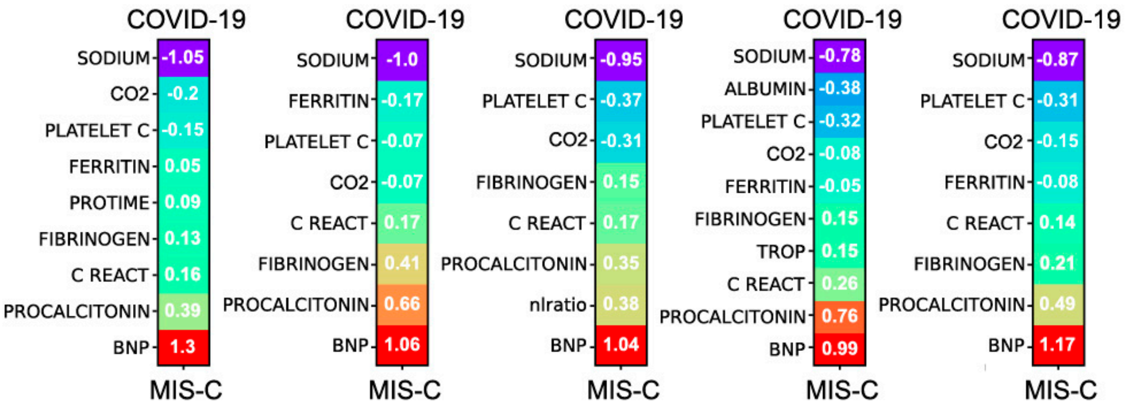

Supplementary Figure S4a: Sorted heat map of 45 measured cytokines/chemokines for the MIS-C and COVID-19 patients in the training set. Each column represents the chemokine/cytokine profile for a patient. The color gradient represents the standardized values of each of the cytokines/chemokines. Patients to the left of the red vertical line are COVID-19 patients, while those to the right are MIS-C. The MIS-C cohort has elevated levels of cytokines/chemokines on average, although the COVID-19 cohort also has patients with elevated levels at the right end. The cytokines/chemokines on the y-axis are sorted in ascending order based on the p-value of the Wilcoxon-rank-sum test to differentiate COVID-19 samples from MIS-C. The horizontal red line demarcates those cytokines/chemokines whose p-value falls above 0.05.

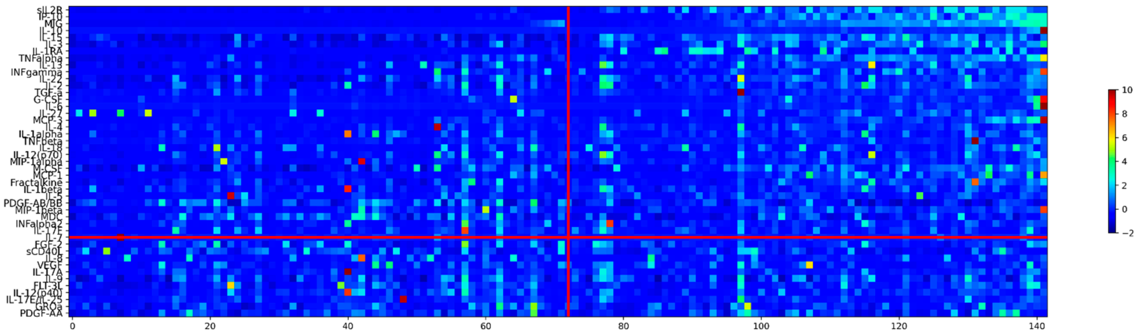

Supplementary Figure S4b: Top: Sorted heat map of 45 measured cytokines/chemokines for the MIS-C and COVID-19 patients in the training set, with classification errors made by the model on the training set itself. Each column represents the chemokine/cytokine profile for a patient. The color gradient represents the standardized values of each of the cytokines/chemokines. Patients to the left of the red vertical line are COVID-19 patients, while those to the right are MIS-C. Note that all seven errors are MIS-C patients with low inflammation levels. **Bottom:** The classification errors in the training set projected into the UMAP space. Note that all seven errors are MIS-C patients, and that six of them map into the COVID-19 clusters, indicating lower than expected inflammation levels, resembling COVID-19 rather than MIS-C.

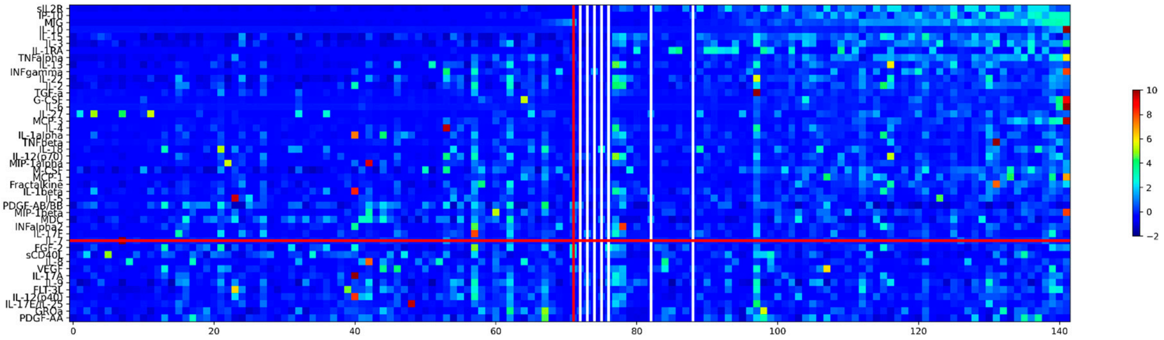

Supplementary Figure S5: ROC curves and confusion matrix for the training cohort, as well as the three validation cohorts.

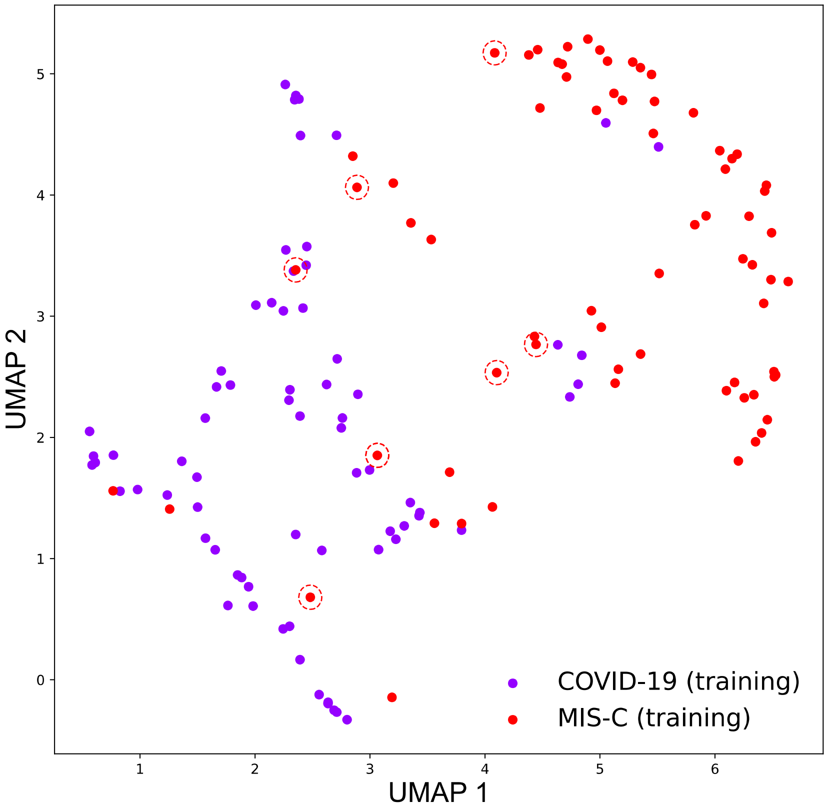

Supplementary Figure S6: Flow chart summary.

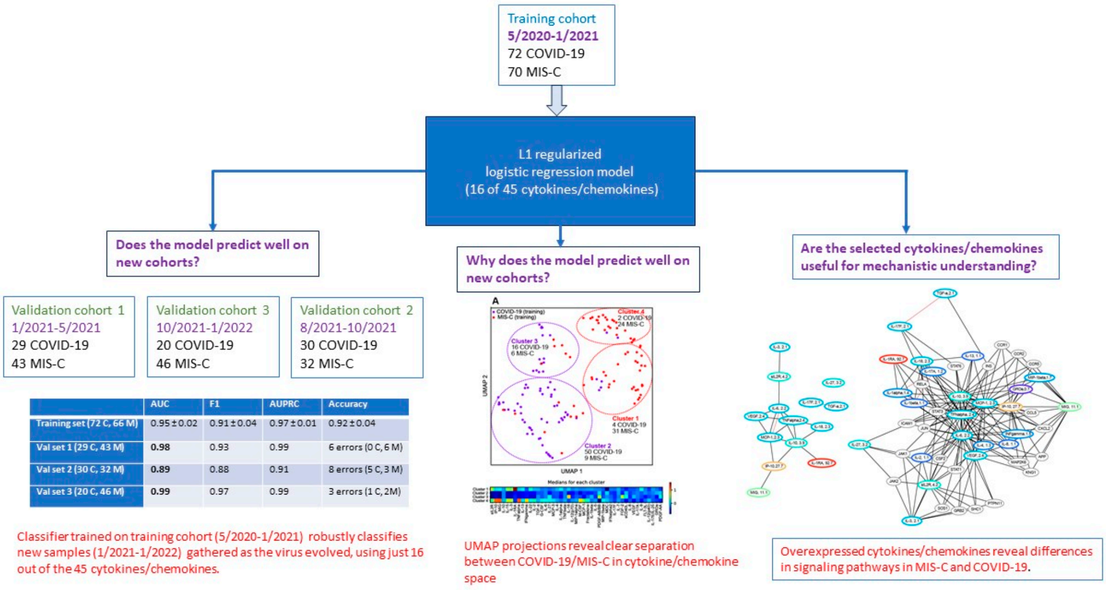

	AUC	F1	AUPRC	Accuracy
Training set (72 C, 66 M)	0.95 ± 0.02	0.91 ± 0.04	0.97 ± 0.01	0.92 ± 0.04
Val set 1 (29 C, 43 M)	**0.98**	0.93	0.99	6 errors (0 C, 6 M)
Val set 2 (30 C, 32 M)	**0.89**	0.88	0.91	8 errors (5 C, 3 M)
Val set 3 (20 C, 46 M)	**0.99**	0.97	0.99	3 errors (1 C, 2M)

Inclusion criteria: age 18 and under, treated at the TCH system for COVID-19, who consented to providing serum samples.
Exclusion criteria: pregnant, having other inflammatory conditions (such as HLH, Kawasaki, sepsis) not related to SARS-Cov-2.

Supplementary Table S1: Performance of logistic regression model trained on laboratory biomarkers of the initial cohort and tested on three *denovo* validation sets.

	AUC	F1	AUPRC	Accuracy
**Training set (72 C, 66 M)**	0.86 ± 0.05	0.78 ± 0.07	0.88 ± 0.06	0.81 ± 0.06
**Val set 1 (29 C, 43 M)**	0.85	0.81	0.89	15 errors (5 C,10 M)
**Val set 2 (30 C, 32 M)**	0.84	0.75	0.84	14 errors (3 C,11 M)
**Val set 3 (20 C, 46M)**	0.83	0.71	0.86	16 errors (4 C, 12 M)

Supplementary Table S2: Characterizing cytokine/chemokine derived UMAP clusters by lab and hospital data. Medians and interquartile ranges are shown for the lab markers. Cluster 2 and Cluster 3 contain predominantly COVID-19 patients, while Cluster 1 and Cluster 4 are primarily MIS-C.

	Cluster 1	Cluster 2	Cluster 3	Cluster 4
**CRP**	7.3 (2.6,18.1)	3.2 (0.9,7.4)	6.3 (0.8,16.9)	16.4 (4.3,22.2)
**Procalcitonin**				
**D-Dimer**	2.1 (1.3,3.5)	1.3 (0.6,2.6)	1.7 (0.5,3.9)	3.9 (2.1,5.8)
**BNP**	109.4 (50.0,342.9)	63.8 (17.9,68.3)	66.8 (29.6,138.7)	300.1 (65.0, 706.2)
**Sodium**	134 (132,139)	138 (135,140)	136.5 (133.3,138)	134.5 (131.3, 137)
**Platelet counts**	207 (119.5,277.5)	243 (161.5,396)	181 (149.8,313.5)	166 (144.3,241)
**Albumin**	3.3 (2.9,3.8)	4.0 (3.4,4.6)	3.5 (3.2,4.2)	3.4 (3.1,3.6)
**Fibrinogen**	458 (383.8, 558)	406.3 (325,535.5)	449 (389.5,596)	485 (385.9,583.8)
**Protime**	15.3 (14.2,16.5)	14.8 (14,15.2)	14.9 (14.7,16.1)	15.3 (14.5,16.2)
**NL ratio**	9.1 (3.5,14.7)	2.4 (1.3,5.4)	5.3 (2.7,11.0)	6.1 (1.9,14.6)
**CO_2**	24 (21,28)	26 (23,27)	26 (24,27.8)	23 (20,26)
**Ferritin**	327.2 (159,485.5)	254.0 (117.5,379.7)	324.4 (147,507.2)	242 (124.3,649.0)
**Troponin I**	0.04 (0.01, 0.11)	0.02 (0.01, 0.07)	0.02 (0.01,0.07)	0.02 (0.01,0.07)
**Length of stay (days) med**	7.9	6.7	5.2	7.82
**ICU LOS (days) median**	3.7	0	2.4	4.47
**ECMO (%)**	0.0	2.1	0.0	10.7
**CPAP (%)**	11.1	17.0	25.0	32.1
**Ventilator (%)**	8.3	23.4	33.3	35.7

	Cluster 1	Cluster 2	Cluster 3	Cluster 4
**AKI (%)**	11.1	12.8	45.8	35.7
**IVIg/steroids 7dys (%)**	38.9	34.0	50.0	39.3
